# The Impact of Heat Treatment on Porcine Heart Valve Leaflets

**DOI:** 10.1007/s13239-017-0334-x

**Published:** 2017-11-13

**Authors:** R. Glenn Hepfer, Peng Chen, Kelvin G. M. Brockbank, Alyce L. Jones, Amanda K. Burnette, Zhen Chen, Elizabeth D. Greene, Lia H. Campbell, Hai Yao

**Affiliations:** 1 Department of Bioengineering, Clemson-MUSC Bioengineering Program, Clemson University, 173 Ashley Avenue, MSC 508, Charleston, SC 29425 USA; 2 Tissue Testing Technologies LLC, North Charleston, SC USA; 3 LifeNet Health, Virginia Beach, VA USA

**Keywords:** Heart valve, Heat treatment, Tissue banking, Cell viability, Collagen content, Matrix permeability

## Abstract

The purpose of this study was to determine the impact of elevated temperature exposure in tissue banking on soft tissues. A secondary objective was to determine the relative ability of various assays to detect changes in soft tissues due to temperature deviations. Porcine pulmonary heart valve leaflets exposed to 37 °C were compared with those incubated at 52 and 67 °C for 10, 30 and 100 min. The analytical methods consisted of (1) viability assessment using the resazurin assay, (2) collagen content using the Sircol assay, and (3) permeability assessment using an electrical conductivity assay. Additionally, histology and two photon microscopy were used to reveal mechanisms of cell and tissue damage. Viability, collagen content, and permeability all decreased following heat treatment. In terms of statistical significance with respect to treatment temperature, cell viability was most affected (*p* < 0.0001), followed by permeability (*p* < 0.0001), and then collagen content (*p* = 0.13). After heat treatment, histology indicated increased apoptosis and two photon microscopy revealed a decrease in collagen fiber organization and an increase in elastin density. These results suggest that measures of cell viability would be best for assessing tissues where the cells are alive and that permeability may be best where cell viability is not intentionally maintained.

## Introduction

Transplant of soft tissue for reconstructive surgery drives the need for processing methods that should have minimal impact on tissue structure and viability. Processing of cryopreserved tissue involves exposure to various temperatures both before and after storage. Furthermore, accidental exposure to elevated temperatures is possible in the donor before procurement, after donation, as well as during graft processing, graft thawing, and rinsing in the operating room. Successful development of processing methods requires sensitive tests to both understand and prevent potential damage from heat exposure. We have previously examined the sensitivity of various analytical tests following a collagenase damage model on porcine pulmonary heart valves.^13^ Here, we expose soft tissues to elevated temperature and test the ability of various assays to detect the change in the tissue. Porcine pulmonary heart valve leaflets were employed as a target tissue, but the general principles can be applied to all soft tissues.

Heart valve leaflets are organized in defined layers: fibrosa, spongiosa and ventricularis, which are composed of different ECM components such as collagen, elastin and proteoglycans.^14^,^23^ The interplay of these layers is responsible for the correct opening and closure of the leaflets and their biomechanical strength and integrity. Modifications of the structure may compromise the mechanical responsiveness of the leaflet eventually leading to failure of aortic or pulmonary valve function. The collagen and elastin exist in a hydrated gel composed of proteoglycans. Collagen and elastin are wavy fibers that can straighten under small loads, allowing large extension of the tissue at low stress. Hydrated elastin is viscoelastic, demonstrated by creep or stress relaxation tests, due to the interaction of elastin molecules with water molecules. Stiffening of elastin occurs by loss of water content.^8^ High level tensile properties of leaflets are entirely dominated by the functionally elastic behavior of the collagen and elastin fiber networks. However, proteoglycans have a pronounced effect at low strains where the collagen is highly crimped.^4^ Since elastin has a denaturation temperature of about 200 °C, which is much higher than collagen’s denaturation temperature of about 65 °C,^6^,^7^,^21^,^24^ this study focused on temperature effects on collagen.

In this study, the effects of heat treatment on porcine heart valves with a variety of analytical tests were studied. The goal was both to reveal mechanisms in which heat damage may compromise the function of transplanted heart valves as well as measure the ability of these tests to detect damage. Leaflet cell viability, collagen content, and a tissue permeability assay based upon electrical conductivity were assessed. Viability is a traditional evaluation of cryopreservation methods for human allograft heart valves. The collagen assay employed in this study is a well-established method for measuring collagen content in mammalian soft tissues. We have previously used the electrical conductivity technique to test the effect of mild enzyme damage on heart valves.^13^ It was found that permeability was impacted more significantly than biomechanics following collagenase exposure. In this study, the conductivity method against cell viability and collagen content were compared. It was hypothesized that the conductivity method would be able to detect heat damage and would offer insights into the mechanism of damage. The mechanisms of cell death were also examined through histology, and changes in collagen and elastin structure were observed through two-photon microscopy.^3^,^12^

## Materials and Methods

### Specimen Preparation

**Figure 1:**
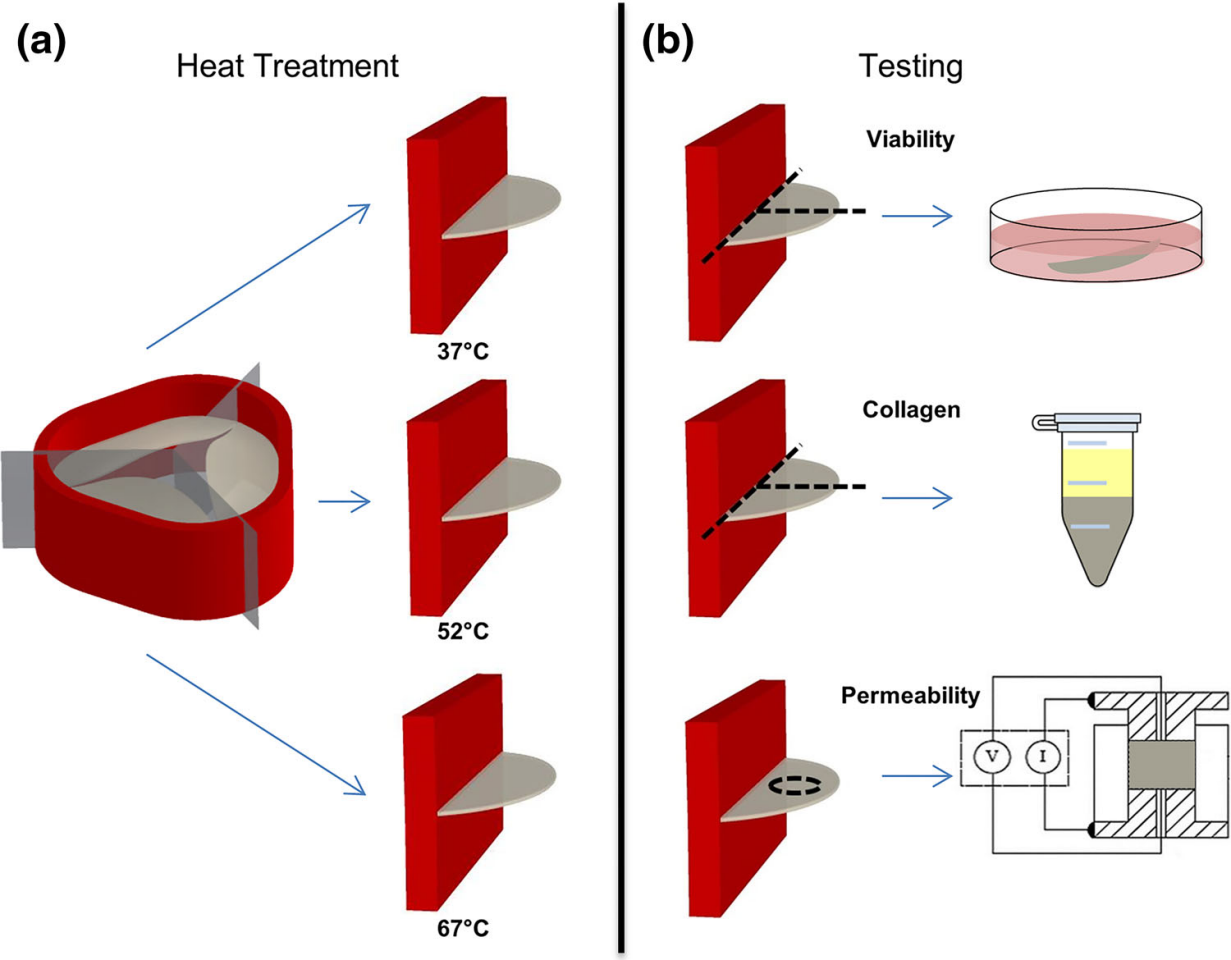
Schematic of sample preparation for each test. Artery is in red and the attached leaflets are in gray. (**a**) Leaflets were exposed to 37, 52, and 67 °C for 10, 30, and 100 min. (**b**) Black dotted lines show how tissue was dissected for testing. For viability and collagen assays, intact or bisected leaflets were used as shown. For the permeability assay, a 5 mm diameter punch was taken from the central region of the leaflet.

Porcine hearts were obtained from an abattoir. A total of 40 pig hearts were procured from a local slaughter house following the current standards of the American Association of Tissue Banks for human cardiac tissue recovery, ischemia and preservation.^20^ The hearts were rinsed and transported on ice in Lactated Ringer’s solution. The pulmonary heart valves were then dissected under aseptic conditions, treated with antibiotics by means of Dulbecco’s modified Eagle medium containing 4.5 g/L glucose (DMEM; Mediatech, Herndon, Va., USA), plus 100 U/mL Penicillin and 100 *μ*g/mL Streptomycin (Sigma, St. Louis, Mo., USA) at 4 °C overnight. The valve leaflets with a section of artery connected by a muscle band were dissected from each valve (Fig. 1). Pulmonary heart valve leaflets were exposed to three temperatures in DMEM: the physiological temperature of 37 °C, the possible temperature of accidental exposure during graft processing of 52 °C,^11^ and the temperature of 67 °C that is just above the denaturation temperature of collagen and is expected to cause significant damage. In addition, an untreated control of fresh tissue was included for reference in the TUNEL assay. Various exposure times of 10, 30, and 100 min were also tested to reveal any danger in failing to properly synchronize thawing procedures with surgery. Bisected or intact individual leaflets were used depending upon the amount of tissue needed for each assay (Fig. 1). Cell viability, collagen content, and permeability assays were then performed.

### Viability Assay

Viability assessment employed the resazurin assay to measure metabolic activity as previously described.^2^, ^19^ The resazurin assay incorporates a water-soluble fluorometric viability oxidation–reduction indicator which detects metabolic activity by both fluorescing and changing color in response to chemical reduction.^19^ Tissue samples were incubated for 3 h with resazurin working solution at 37 °C, and aliquots of medium were then placed in microtiter plate wells and read on a microtiter plate spectrofluorometer at a wavelength of 590 nm. The results were normalized as relative fluorescent units (RFU)/mg dry weight after subtraction of background florescence. Tissue was lyophilized for 24 h for calculation of dry weight. A total of 54 (6 samples for each of the 9 treatment groups) bisected or intact individual leaflets were used for the viability assay. Since the test is non-cytotoxic and non-destructive, for each sample the assay was performed on days 0, 1, 2, and 3 of culture.

### TUNEL Assay

Tissue samples for histology were fixed, sectioned, stained with hematoxylin and eosin (H&E) and by the *in situ* terminal transferase mediated dUTP nick-end labeling (TUNEL) method for detection of apoptotic cells. Digital photographs were made using a light microscope. Morphometric analysis of TUNEL stained sections was performed by manual counting of affected cells in representative photographs and normalized by total cell count.

### Sircol Collagen Assay

The Sircol Collagen Assay^1^ is a quantitative dye-binding method designed for the analysis of acid‐soluble collagens extracted from mammalian tissues and collagens released into culture medium by mammalian cells during *in vitro* culture (Biocolor Ltd., Newtownabbey, Northern Ireland). The Sircol dye reagent contains Sirius Red (Direct Red 80). Sirius Red is an anionic dye with sulphonic acid side chain groups that react with the side chain groups of the basic amino acids present in collagen. The specific affinity of the dye for collagen, under the assay conditions, is due to the elongated dye molecules becoming aligned parallel to the long, rigid structure of native collagens that have intact triple helix organization. Dye affinity is significantly reduced when collagen is heat denatured to form of random chains of gelatin and the triple helix unwinds. The amount of acid-pepsin soluble collagen in leaflet samples was quantified per manufacturer’s instructions by measuring the binding and resultant absorbance of Sirius Red at 555 nm. A total of N = 54 samples (6 samples for each of the 9 treatment groups) were tested for collagen content.

### Permeability Assay

For permeability assessment, 5 mm diameter punch biopsies were taken from the central region of intact leaflets. Measurements were performed in isotonic PBS solution using an electrical conductivity assay as previously described.^2^ The electrical conductivity was measured based on the principle of a four wire resistance test using a Keithley Sourcemeter (Model 2400, Keithley Instruments, Inc., Cleveland, OH) and a custom designed conductivity chamber reported previously.^2^,^9^ Briefly, the conductivity apparatus consists of two stainless steel current electrodes coaxial to two Teflon-coated Ag/AgCl voltage electrodes placed on the top and bottom of a cylindrical nonconductive Plexiglass chamber (5 mm diameter). The specimen was placed inside the chamber for measurement. The resistance (*R*) values across the specimens were measured at a low, constant DC current density of 0.015 mA/cm.^1^ The height of the specimen was measured with an electrical current sensing micrometer. The electrical conductivity (χ) values of the specimens were calculated by $\chi = \frac{h}{R \cdot A}$ where *h* and *A* are the height and cross-sectional area of the specimens, respectively. The precision for the resistance measurements was 0.5 Ω while the height measured with an accuracy of ± 1.0 *μ*m. All electrical conductivity measurements were performed at room temperature (22 °C). A total of *N* = 54 samples (6 samples for each of the 9 treatment groups) were used. For each sample, conductivity was calculated in forward and reverse directions and the average of these two was reported.

### Two-Photon Imaging

The central region of the outflow (adventitia) side of intact leaflets was imaged using second-harmonic generation (SHG) and two-photon emission fluorescence (TPEF) following 10 min treatments at 37, 52, and 67 °C. The leaflets were mounted on a 35 mm glass bottom dish (MatTek, Ashland, MA). The heart valves were never stained or sectioned prior to imaging. Imaging was performed using an Olympus Fluoview 1200 MPA (Olympus, Center Valley, PA), with a 30 × oil immersion objective lens (UPLSAPO, 30XSIR; Olympus, Center Valley, PA). For SHG, the excitation laser was at a wavelength of 860 nm, and the signal was collected in the 420–460 nm range. For TPEF, the excitation laser was at a wavelength of 780 nm and the signal was collected in the 520–600 nm range. Image resolution was 1024 × 1024 with a field of view of 423 × 423 *µ*m.^28^,^30^

### Statistics

The samples for cell viability, collagen content, permeability, and histology were blinded so that the test operator could not discern between samples. The data were statistically evaluated by analysis of variance (ANOVA) and Tukey’s *post hoc* tests using SAS software, version 9.2 for XP-Pro (Cary, NC, USA). Statistical differences were reported at *p*-values < 0.05. The results were reported as mean ± standard error.

## Results

### Viability Assay

**Figure 2:**
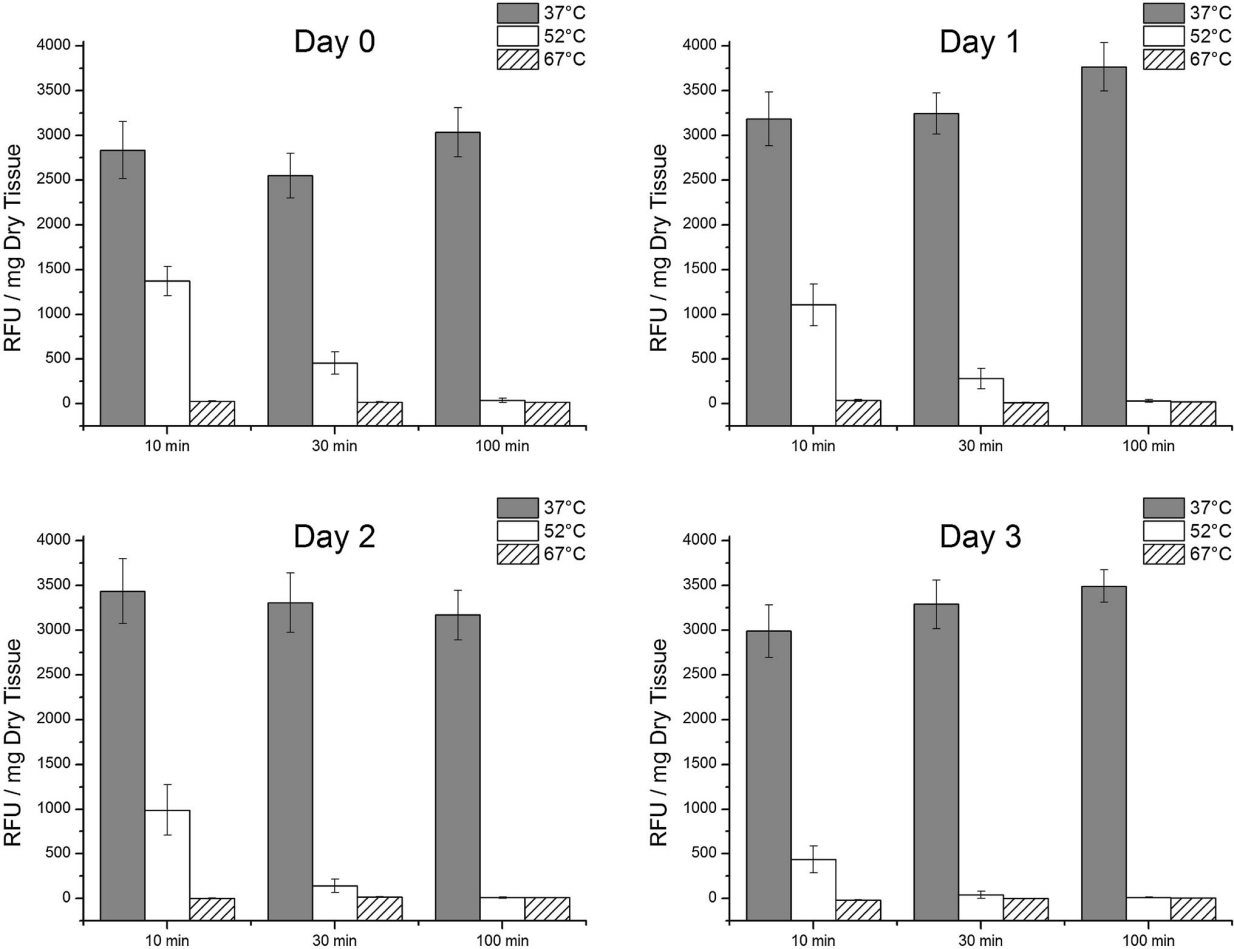
Metabolism assay for heart valves, with standard error bars, of different heat treatment temperatures and times cultured for 0, 1, 2, and 3 days. *N* = 6 for all groups. Two-way ANOVA: Temperature groups were significantly different for all days of culture (*p* < 0.0001). Time groups were significantly different on day 0 of culture (*p* = 0.01). *Post-hoc* Tukey: for day 0 and day 1, the 37, 52, and 67 °C groups were all significantly different from each other (*p* < 0.01); for day 2 and day 3, the 52 and 67 °C groups were significantly different from the 37 °C (*p* < 0.01); for day 0, the 30 min and 100 min groups were significantly different from the 10 min group (*p* < 0.05).

The general trends were a decrease in viability with increasing incubation temperature, incubation time, and day of culture (Fig. 2). At Day 0 of culture, for 10 min of incubation time, average viabilities for 37, 52, and 67 °C of incubation temperature were 2835 ± 318, 1371 ± 162, and 27 ± 3 RFU/mg of tissue, respectively. For 30 min of incubation, viabilities were 2550 ± 249, 457 ± 123, and 18 ± 2 RFU/mg of tissue. For 100 min of incubation, viabilities were 3035 ± 274, 38 ± 26, and 17 ± 1 RFU/mg of tissue. The difference between temperature groups were consistently significant for each day of culture (*p* < 0.0001) while the difference between time groups was only significant on day 0 of culture (*p* = 0.01) by two-way ANOVA. With respect to the effect of culture day on viability, after elevated heat treatment (52 and 67 °C), viability generally decreased with increased day of culture. However, for the samples incubated at 37 °C, viability seemed to either not change or increase with increased day of culture. For all groups, N = 6.

### TUNEL Assay

**Figure 3:**
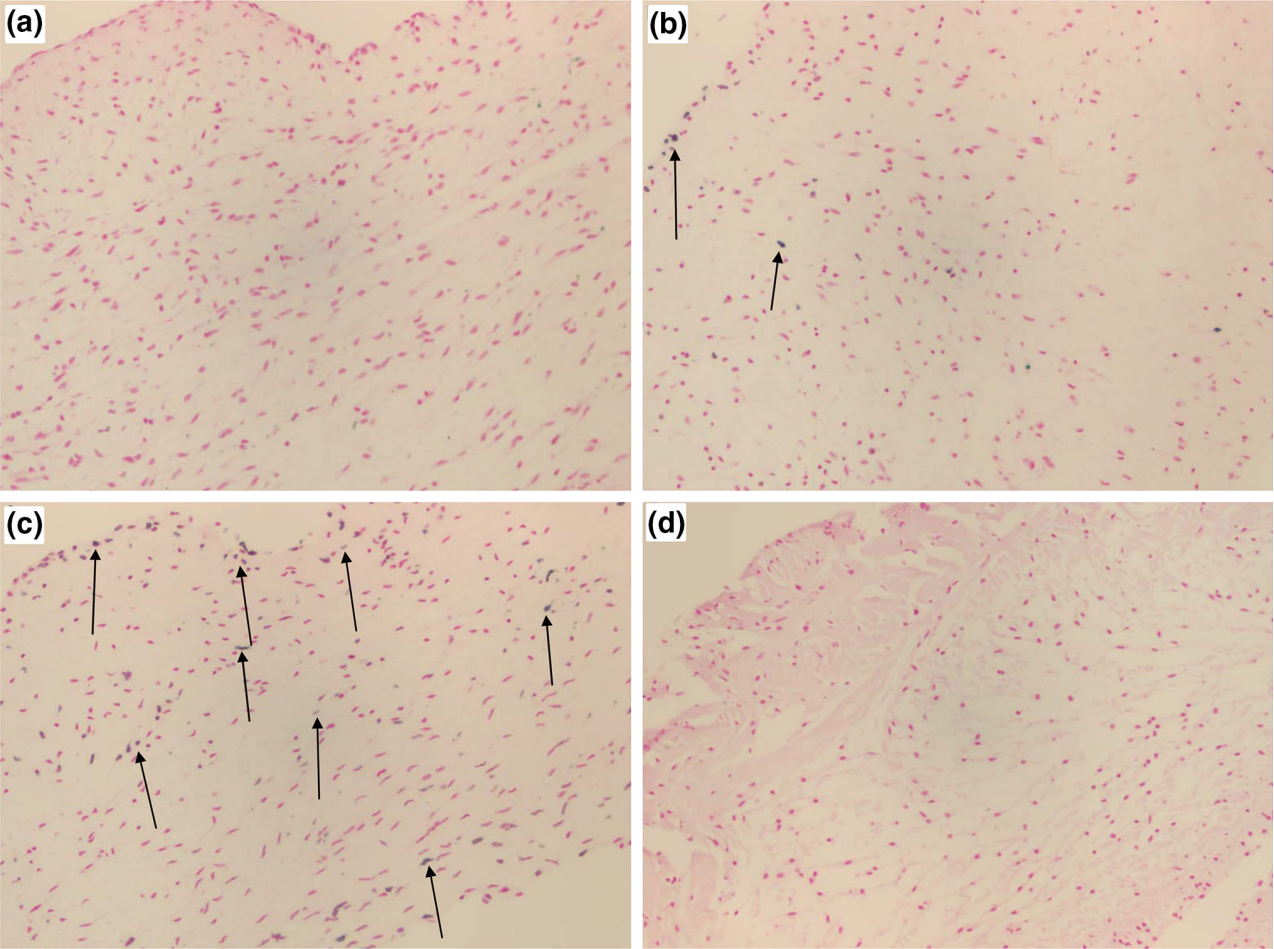
Hematoxylin and Eosin and TUNEL staining on heart valve leaflets. (**a**) Untreated control. (**b**) 37 °C for 100 min. (**c**) 52 °C for 100 min. (**d**) 67 °C for 10 min. Arrows point to TUNEL positive cells stained blue. No TUNEL positive cells were observed at any time point for 67 °C because all the cells died by necrosis.

Percent of apoptotic cells increased *in vitro* during 100 min of 52 °C heat treatment (Table 1). The decreases in cell viability were associated with elevated levels of apoptosis detected by the TUNEL stain (Fig. 3). After 100 min at 52 °C, 64 ± 22% apoptotic cells were observed compared with 6 ± 4% in samples treated at 37 °C for 100 min. The group treated at 37 °C demonstrated no significant changes during 100 min of incubation *in vitro* when compared to untreated controls. No viable cells or TUNEL positive cells were observed after ≥10 min of heat treatment at 67 °C. For each treatment group, two slides were prepared and three regions of interest were imaged for each slide.

**Table 1:** Average percentage of cells showing apoptosis via TUNEL staining with standard errors of the mean.

Treatment	Untreated control	37 °C (100 min)	52 °C (10 min)	52 °C (100 min)	67 °C (10 min)
% Apoptotic cell	3 ± 3	6 ± 4	12 ± 8	64 ± 22	0 ± 0

For each treatment group, two slides were prepared and three regions of interest were imaged for each slide

### Sircol Collagen Assay

**Figure 4:**
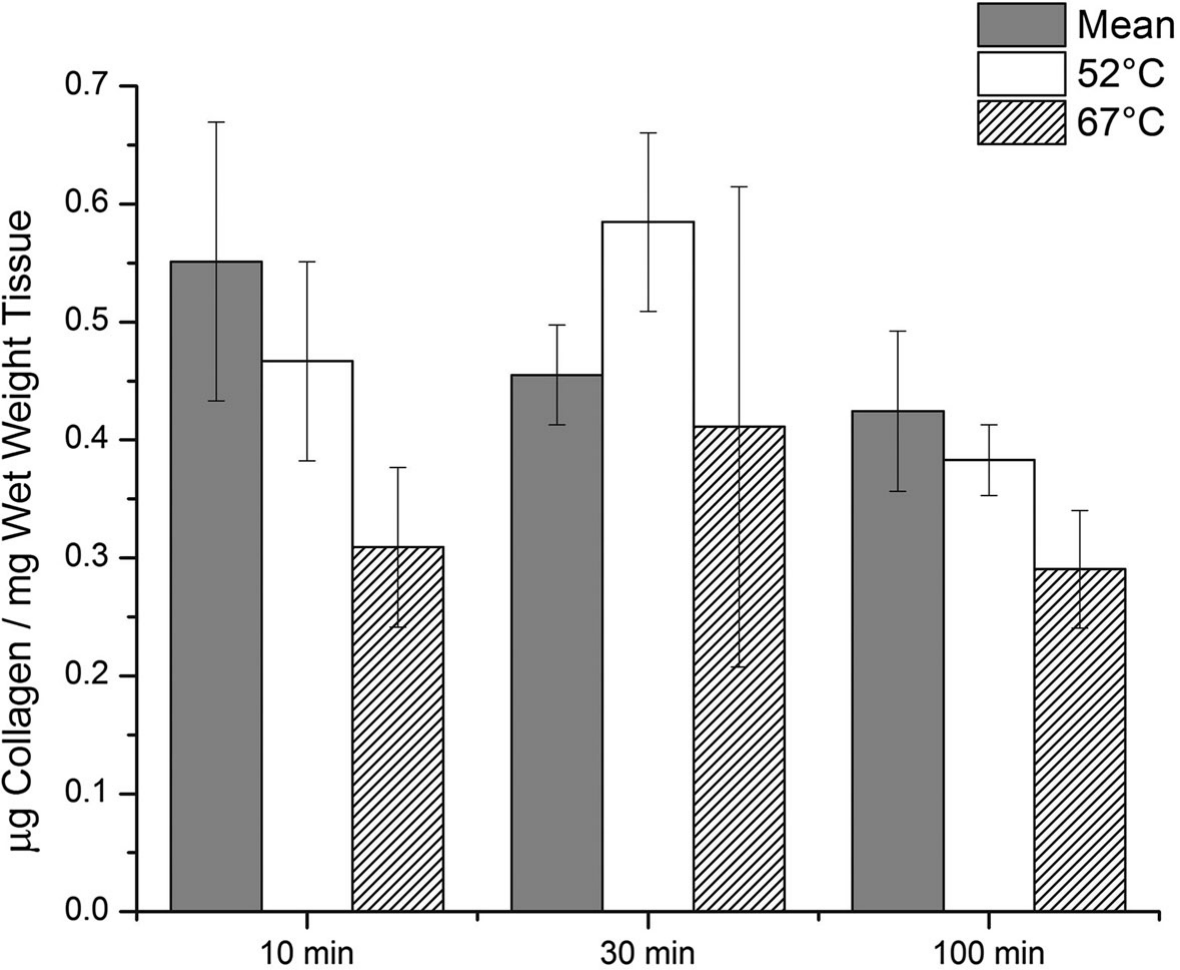
Collagen content for heart valves, with standard error bars, for all heat treatment temperatures and times. Two-way ANOVA: No significant difference was found between temperature groups (*p* = 0.13) or time groups (*p* = 0.32).

The trend was for a decrease in collagen content with increasing incubation temperature and time (Fig. 4). For example, for 10 min of incubation time, average collagen contents for 37, 52, and 67 °C of incubation temperature were 0.55 ± 0.12, 0.47 ± 0.08, and 0.31 ± 0.07 *μ*g/mg wet tissue, respectively. However, neither the difference between temperature groups (*p* = 0.13) nor the difference between time groups (*p* = 0.32) was statistically significant by two-way ANOVA. For all groups, *N* = 6.

### Permeability Assay

**Figure 5:**
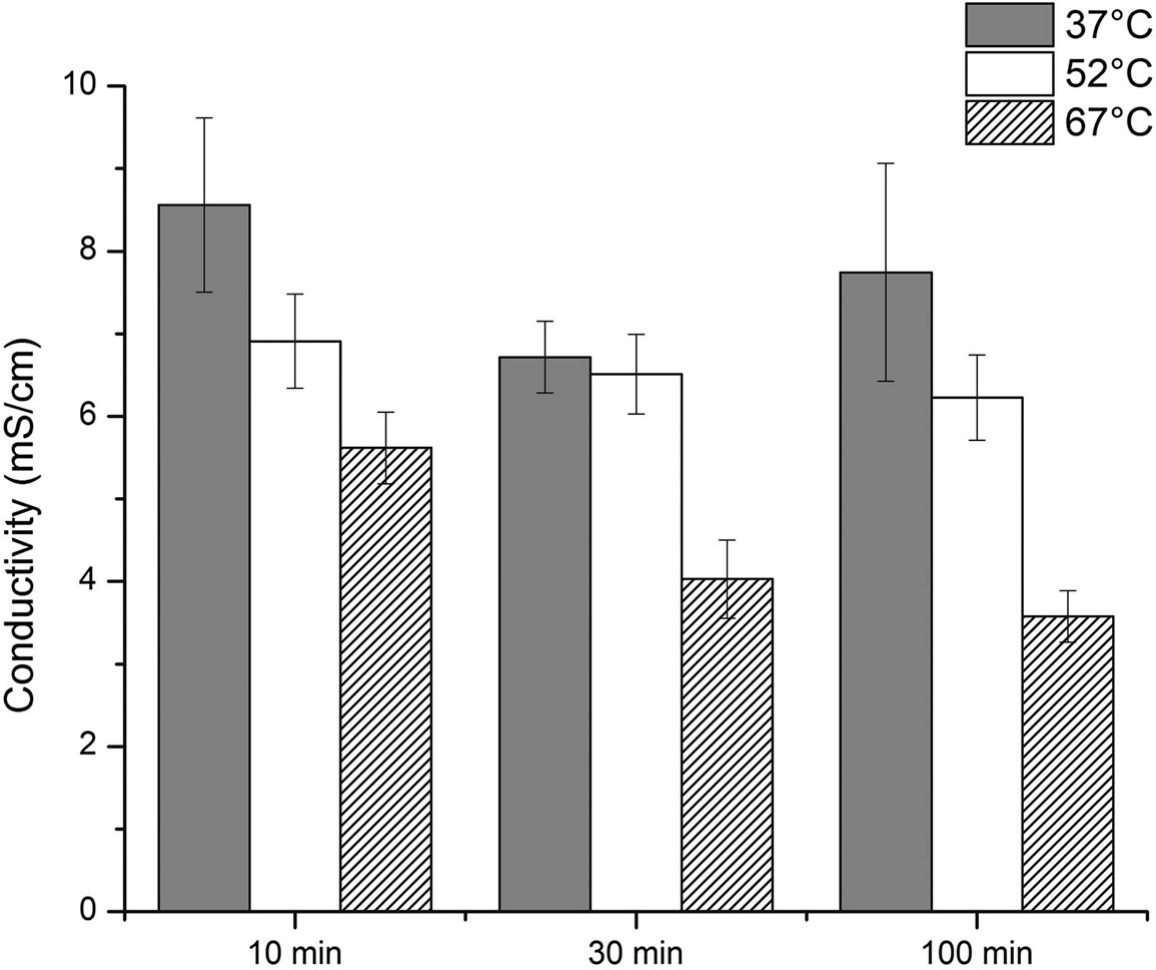
Conductivity and standard error bars of heart valves for all heat treatment temperatures and times. Two-way ANOVA: The difference between temperature groups was significant (*p* < 0.0001) while the difference between time groups approached significance (*p* = 0.05). *Post-hoc* Tukey: the 37 °C (*p* < 0.0001) and 52 °C (*p* = 0.001) groups were significantly different from the 67 °C group.

Permeability assessment using electrical conductivity, shown in Fig. 5, demonstrated decreased conductivity with increased incubation temperature. The trend was also for decreased conductivity with increased incubation time. For 10 min of incubation time, average conductivities for 37, 52, and 67 °C of incubation temperature were 8.56 ± 1.06, 6.91 ± 0.57, and 5.62 ± 0.43 mS/cm, respectively. For 30 min, conductivities were 6.72 ± 0.44, 6.51 ± 0.48, and 4.03 ± 0.47 mS/cm. For 100 min, conductivities were 7.74 ± 1.32, 6.23 ± 0.52, and 3.58 ± 0.31 mS/cm. The difference between temperature groups were significant by two-way ANOVA (*p* < 0.0001) while the difference between time groups approached significance (*p* = 0.05). The decrease in conductivity after incubation at 67 °C was statistically significant when compared to the conductivity after incubation at 37 °C (*post hoc* Tukey’s, *p* < 0.0001) as well as to the conductivity after incubation at 52 °C (*post hoc* Tukey’s, *p* = 0.001). For all groups, *N* = 6.

### Two-Photon Imaging

**Figure 6:**
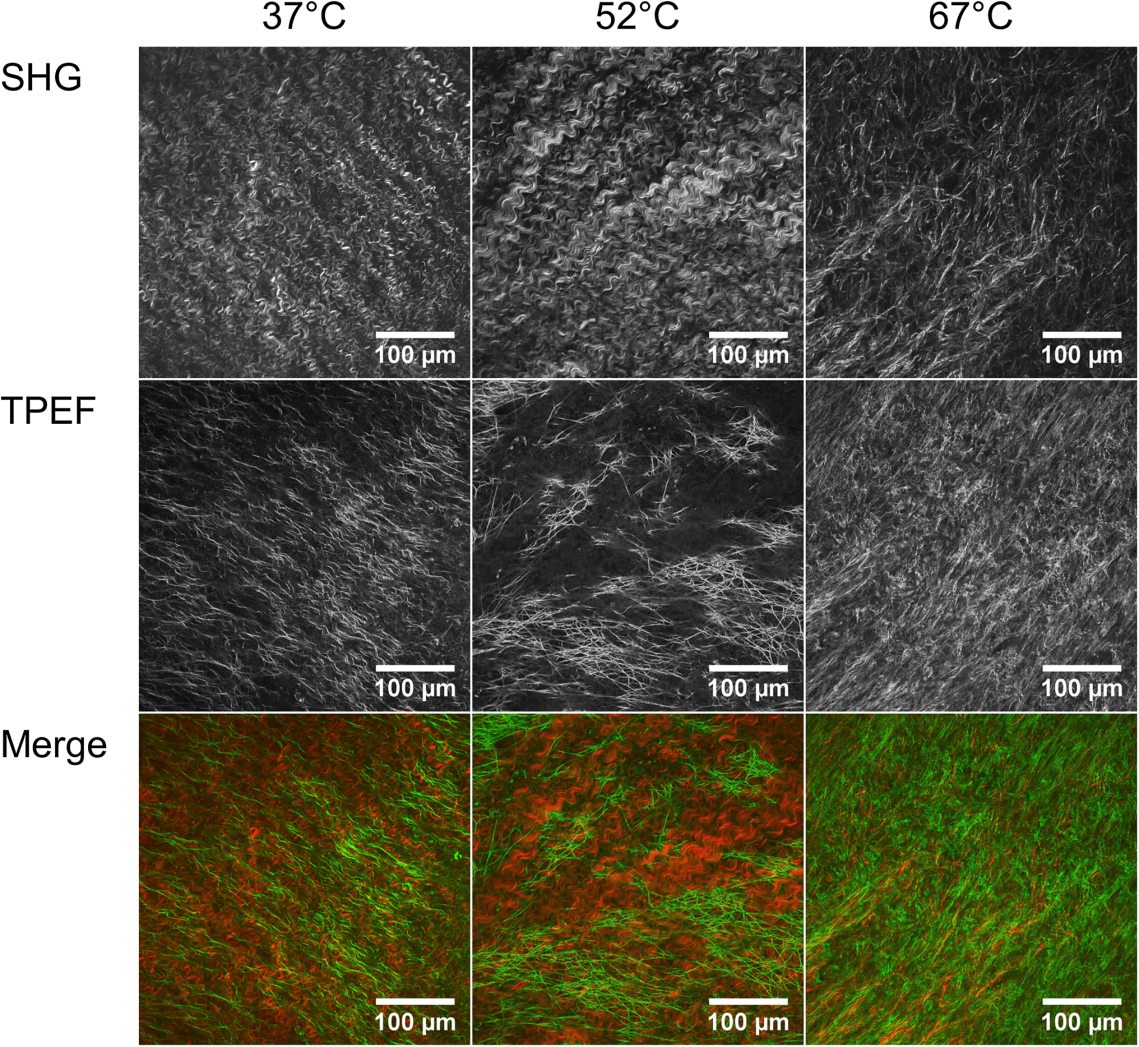
Second harmonic generation (SHG) and two-photon emission fluorescence (TPEF) of heart valves treated at 37, 52, and 67 °C for 10 min. SHG shows collagen while TPEF shows elastin. In the merged file collagen is colored red and elastin green.

Collagen structure revealed by second harmonic generation is well organized and exhibits a high degree of crimping or waviness of well-defined bundles in the heart valve leaflets treated at 37 and 52 °C (Fig. 6). By contrast, collagen structure of the leaflet treated at 67 °C exhibits much less organization or alignment. The two-photon emission fluorescence shows long interwoven strands of elastin in the leaflets treated at 37 and 52 °C. In the valve treated at 67 °C, the TPEF signal is increased and the elastin appears denser (Fig. 6).

## Discussion

Pulmonary allograft heart valves are the most commonly transplanted allograft heart valve^17^,^27^ and rely on an intricate collagen and elastin structure to function properly. These transplants are particularly important in pediatric patients that are still developing. In this study, we evaluated the effect of heat damage on porcine pulmonary valve leaflets with some common tests as well as a novel conductivity test. The heat treatment model simulates damage that could occur due to accidental exposure to heat during preparation of allograft heart valves for implantation. The tissue banking industry employs standard tests in developing processing methods that minimize tissue damage. There is a need to verify the sensitivity of these tests. While we use pulmonary valves in this study, the heat treatment could easily be applied to allograft aortic valves or other soft tissues such as tendons and used in experimental or process validation studies as controlled tissue damage.

Our results showed that viability was most significantly affected by heat damage. Viability is a traditional evaluation of cryopreserved human allograft heart valves being used for transplant, as it is a marker for overall valve biocompatibility. Viability may be defined as the relative ability of cells or tissues to perform their normal metabolic functions. Many means of assessing cell viability have been described for heart valve tissues including amino acid uptake, protein synthesis, contractility, dye uptake, ribonucleic acid synthesis, 2-deoxyglucose phosphorylation and the resazurin metabolism assay.^13^ The metabolism-based resazurin assay^19^ is non-cytotoxic and non-destructive. The same heart valve leaflet can be assayed several times if it is maintained under physiological tissue culture conditions. However, care should be taken interpreting the results of individual assays such as the resazurin assay. In a previous study of heart valve leaflets after collagenase treatment the resazurin assay demonstrated increases in metabolic activity with duration of mild collagenase treatment compared with untreated controls.^13^ A likely hypothesis to explain the observed increase was collagen damage leading to higher water content and improved permeability and diffusion of nutrients or viability assay reagent. An observed increase in permeability via the electrical conductivity method in this previous study supports this hypothesis. On the other hand, multiple time point assessment using the resazurin assay can identify process effects on the tissue’s cells that can then be assessed using more specific assays such as the TUNEL stain for apoptotic cells. In this case, decreases in cell viability (Fig. 2) were associated with elevated levels of apoptosis (Table 1; Fig. 3). Cells in 67 °C treated tissues were dead, demonstrating no delayed cell death.

The Sircol Assay is a dye-binding method designed for the analysis of acid soluble collagen and pepsin soluble collagen by absorbance readings within the range 520–570 nm. This assay is a well-established method for measuring collagen content of mammalian soft tissues and has been employed for decades.^1^,^22^ While we observed a distinct trend of decreasing collagen content with increasing incubation temperature and time, the data failed to achieve statistical significance (Fig. 4). Of all the methods evaluated in this study, the collagen assay showed the least impact due to heat exposure. Collagen content appears to not be as severely affected by heat exposure compared to cell viability or permeability.

Electrical conductivity of biological tissues is related to the diffusivity of small ions in the tissue, which depends upon tissue composition and structure.^5^,^18^ The effect of matrix composition on solute permeability has previously been studied in hydrogels and cartilaginous tissues using an electrical conductivity method.^10^,^15^,^16^ These studies demonstrated that electrical conductivity is positively correlated with tissue porosity (i.e., water volume fraction). Therefore, the decreased conductivity observed after heat treatment was likely due to water loss and increased density of the tissue matrix (Fig. 5). While we observed an insignificant trend of decreasing collagen content (Fig. 4), the TPEF images appear to show an increase in elastin density (Fig. 6). This increased elastin density could contribute to the overall decrease in conductivity, especially since we do not expect elastin to denature at these temperatures. Thus, the conductivity test appears to have detected an important change in functional ECM structure that was not detected with significance by the collagen assay. The mechanical response of tissues to external loading is determined by the interaction between its solid and fluid domains, which translates to the interstitial fluid flow through the porous deformable solid phase.^26^ During the normal cardiac cycle, valve leaflets routinely withstand large deformations with changes in area as high as 50%.^25^ Such observations indicate a significant role of interstitial fluid flow in mechanical function of the leaflet.^29^ Recently, we demonstrated increased conductivity after collagenase treatment of heart valve leaflets that was likely due to increased water content.^13^ These observations using collagenase combine with the present results using heat to suggest that permeability assessment by measurement of conductivity may be a useful tool for assessment of processing steps on soft tissues that is independent of cell viability. Permeability testing promises to provide a sensitive quantitative test that can be employed for validation of process changes for soft tissues.

In conclusion, our results indicate that, of the properties we measured, cell viability would be most affected by accidental exposure to elevated temperatures, followed by permeability and collagen content. The detected damages underscore the importance in designing processing procedures for transplant tissue that prevent accidental heat exposure. The parameters may also be used in designing and testing new preservation methods that minimize tissue damage. Measures of cell viability may be best for assessing tissues where the cells are alive with permeability as an assessment of non-vital tissues or relative to the intended surgical use of the tissue. Furthermore, permeability may be best for tissue processing methods where cell viability is not intentionally maintained such as after tissue decellularization procedures or when tissues are frozen without cryoprotectants. Permeability may be an important indicator of tissue structure and function, even, as seen here, when collagen content does not significantly change. These results underscore the need for comprehensive testing of a variety of parameters in designing tissue processing methods. Heat treatment promises to be an effective model for demonstration that assays have adequate sensitivity to detect changes. This could be of importance for development of new or modified processes by tissue banks.

## References

[1] Anderson SM, Elliott RJ (1991). Evaluation of a new, rapid collagen assay. Biochem Soc Trans.

[2] Brockbank KG, Rahn E, Wright GJ, Chen Z, Yao H (2011). Impact of hypothermia upon chondrocyte viability and cartilage matrix permeability after 1 month of refrigerated storage. Transfus Med Hemother.

[3] Chen X, Nadiarynkh O, Plotnikov S, Campagnola PJ (2012). Second harmonic generation microscopy for quantitative analysis of collagen fibrillar structure. Nat Protoc.

[4] Eckert CE, Fan R, Mikulis B, Barron M, Carruthers CA, Friebe VM, Vyavahare NR, Sacks MS (2013). On the biomechanical role of glycosaminoglycans in the aortic heart valve leaflet. Acta Biomater.

[5] Frank EH, Grodzinsky AJ, Phillips SL, Grimshaw PE, Mow VC, Wood DO, Woo SL (1990). Physiochemical and bioelectrical determinants of cartilage material properties. Biomechanics of Diarthrodial Joints.

[6] Fung YC (1993). Biomechanics: Mechanical Properties of Living Tissue.

[7] Goissis G, Suzigan S, Parreira DR, Maniglia JV, Braile DM, Raymundo S (2000). Preparation and characterization of collagen-elastin matrices from blood vessels intended as small diameter vascular grafts. Artif Organs.

[8] Gosline JM, French CJ (1979). Dynamic mechanical properties of elastin. Biopolymers.

[9] Gu WY, Justiz MA, Yao H (2002). Electrical conductivity of lumbar anulus fibrosis: effects of porosity and fixed charge density. Spine (Phila Pa 1976).

[10] Gu WY, Yao H, Vega AL, Flagler D (2004). Diffusivity of ions in agarose gels and intervertebral disc: effect of porosity. Ann Biomed Eng.

[11] Hazard report update (2009). ECRI Institute revises its recommendation for temperature limits on blanket warmers. Health Devices.

[12] He B, Wu JP, Chim SM, Xu J, Kirk TB (2013). Microstructural analysis of collagen and elastin fibres in the kangaroo articular cartilage reveals a structural divergence depending on its local mechanical environment. Osteoarthr Cartil.

[13] Hepfer RG, Brockbank KG, Chen Z, Greene ED, Campbell LH, Wright GJ, Linthurst-Jones A, Yao H (2016). Comparison and evaluation of biomechanical, electrical, and biological methods for assessment of damage to tissue collagen. Cell Tissue Bank.

[14] Hinton RB, Yutzey KE (2011). Heart valve structure and function in development and disease. Annu Rev Physiol.

[15] Jackson A, Gu W (2009). Transport properties of cartilaginous tissues. Curr Rheumatol Rev.

[16] Kuo J, Wright GJ, Bach DE, Slate EH, Yao H (2011). Effect of mechanical loading on electrical conductivity in porcine TMJ discs. J Dent Res.

[17] Lisy M, Kalender G, Schenke-Layland K, Brockbank KG, Biermann A, Stock UA (2017). Allograft heart valves: current aspects and future applications. Biopreserv Biobank.

[18] Maroudas A (1968). Physicochemical properties of cartilage in the light of ion exchange theory. Biophys J.

[19] O’Brien J, Wilson I, Orton T, Pognan F (2000). Investigation of the Alamar Blue (resazurin) fluorescent dye for the assessment of mammalian cell cytotoxicity. Eur J Biochem.

[20] Osborne JC, Norman KG, Maye T, Malone P, Brubaker SA (2016). Standards for Tissue Banking.

[21] Pezzin G, Scandola M, Gotte L (1976). The low-temperature mechenical relaxation of elastin. I. The dry protein. Biopolymers.

[22] Ramaswamy S, Gottlieb D, Engelmayr GC, Aikawa E, Schmidt DE, Gaitan-Leon DM, Sales VL, Mayer JE, Sacks MS (2010). The role of organ level conditioning on the promotion of engineered heart valve tissue development *in vitro* using mesenchymal stem cells. Biomaterials.

[23] Sacks MS, Schoen FJ, Mayer JE (2009). Bioengineering challenges for heart valve tissue engineering. Annu Rev Biomed Eng.

[24] Samouillan V, Lamure A, Maurel E, Dandurand J, Lacabanne C, Spina M (2000). Dielectric characterization of collagen, elastin, and aortic valves in the low temperature range. J Biomater Sci Polym Ed.

[25] Schoen FJ, Levy RJ (1999). Founder’s Award, 25th Annual Meeting of the Society for Biomaterials, perspectives. Providence, RI, April 28–May 2, 1999. Tissue heart valves: current challenges and future research perspectives. J Biomed Mater Res.

[26] Swartz MA, Fleury ME (2007). Interstitial flow and its effects in soft tissues. Annu Rev Biomed Eng.

[27] VeDepo MC, Detamore MS, Hopkins RA, Converse GL (2017). Recellularization of decellularized heart valves: progress toward the tissue-engineered heart valve. J Tissue Eng.

[28] Wagnieres GA, Star WM, Wilson BC (1998). *In vivo* fluorescence spectroscopy and imaging for oncological applications. Photochem Photobiol.

[29] Wang L, Korossis S, Fisher J, Ingham E, Jin Z (2011). Prediction of oxygen distribution in aortic valve leaflet considering diffusion and convection. J Heart Valve Dis.

[30] Zipfel WR, Williams RM, Christie R, Nikitin AY, Hyman BT, Webb WW (2003). Live tissue intrinsic emission microscopy using multiphoton-excited native fluorescence and second harmonic generation. Proc Natl Acad Sci USA.

